# Effect of exercise based on ACSM recommendations on blood pressure and heart rate in hypertensive patients: A systematic review and meta-analysis of randomized controlled trials

**DOI:** 10.1371/journal.pgph.0003743

**Published:** 2024-12-16

**Authors:** Jiu Chen, Wenlai Cui, Jun Xie

**Affiliations:** Graduate School, Capital University of Physical Education and Sports, Beijing, China; Orthopedic Clinic Orthosport, BOSNIA AND HERZEGOVINA

## Abstract

Numerous studies have demonstrated the positive effects of exercise as a non-pharmacological treatment for hypertensive patients. However, there was a relative lack of research analyzing the effects of different exercise doses on hypertensive individuals. Therefore, the primary objective of this study was to evaluate the impact of different exercise doses on blood pressure (BP) and heart rate (HR) in hypertensive patients through a systematic review and meta-analysis. A systematic search was conducted across four electronic databases (PubMed, Embase, Web of Science, and Cochrane), focusing on the impact of exercise on BP and HR in hypertensive patients, followed by literature screening. Based on the American College of Sports Medicine (ACSM) recommendations for aerobic, resistance, and flexibility exercises in hypertensive patients, the intervention measures from 29 randomized controlled trials were evaluated and categorized as high adherence and low/uncertainty adherence groups according to ACSM recommendations. Differences in systolic blood pressure (SBP), diastolic blood pressure (DBP), and HR between ACSM high and low/uncertainty adherence exercises were reported and evaluated using standardized mean difference (SMD) and 95% confidence interval (95% CI). A total of 25 articles were included, comprising 29 studies, with 16 studies categorized as high adherence with ACSM recommendations and 13 categorized as low or uncertain adherence. For the three outcome measures, the SMD ratio of exercise interventions with high ACSM adherence to those with low or uncertain ACSM adherence was as follows: systolic blood pressure (− 1.20: − 0.75), diastolic blood pressure (− 0.84: − 0.78), and heart rate (− 0.37: − 0.40). The results suggest that exercise interventions with high adherence to ACSM recommendations had a more significant impact on SBP and DBP in hypertensive patients, while the impact on HR was less pronounced than that of interventions with low or uncertain adherence to ACSM recommendations.

This systematic review and meta-analysis was registered in PROSPERO (CRD 42023460293).

## Background

Hypertension as one of the most prevalent chronic diseases is a major risk factor for coronary heart disease, stroke, heart failure, and chronic kidney disease [1–3]. According to the World Health Organization (WHO), hypertension has affected 1 billion people globally, leading to approximately 9 million deaths each year attributed to high blood pressure [4]. It is projected that the number of adults with hypertension will increase by 60%, reaching 1.56 billion by 2025 [5]. To prevent complications associated with hypertension, enhance patients’ quality of life, and ensure patient safety, it is crucial to explore effective prevention and treatment strategies.

Current treatments for hypertension include both pharmacological and non-pharmacological interventions. First-line medications for hypertension include diuretics, beta-blockers, angiotensin-converting enzyme inhibitors, calcium channel blockers, and others [6]. However, studies have shown that approximately 70% of patients who only receive medication were unable to control their condition effectively [7]. Furthermore, long-term medication can be costly and may have associated side effects [8, 9]. As a result, non-pharmacological treatments, especially physical exercise, have gained increasing attention and recognition [10]. Regular physical activity can lower blood pressure in hypertensive patients [11, 12], which in turn can reduce the incidence and mortality of stroke, myocardial infarction, and cardiovascular diseases [13]. The International Guidelines for the Management of Hypertension recommend lifestyle modifications for hypertensive patients, such as adopting healthy eating habits, losing weight, reducing alcohol and salt intake, and engaging in regular physical activity [14–16].

In recent years, numerous meta-analyses have focused on exploring the effects of various forms of exercise on the health of hypertensive patients. Many studies have confirmed the preventive and therapeutic benefits of exercise as a non-pharmacological treatment for hypertension. For example, Igarashi highlighted that different forms of aquatic exercise, such as swimming, water resistance training, and water walking, can lower blood pressure [17]. Yin found that performing Tai Chi more than five times a week for over 12 weeks is more effective in reducing blood pressure and improving lipid metabolism, compared to other Tai Chi exercise cycles for hypertension [18]. Goldberg demonstrated that moderate-intensity aerobic exercise can significantly improve endothelial function and reduce arterial stiffness in hypertensive patients [19]. Zhou indicated that various forms of exercise (aerobic exercise, resistance training, and high-intensity interval training) can significantly increase flow-mediated dilation (FMD) and lower blood pressure in hypertensive individuals [20].

The American College of Sports Medicine (ACSM) provides exercise prescriptions for hypertensive patients, which include recommendations for aerobic, resistance, and flexibility exercises. These prescriptions detail the frequency, intensity, duration, and type of each exercise category [21]. However, there is a relative lack of research analyzing the effects of different exercise doses on hypertensive patients. Therefore, we conducted a systematic review of existing studies to evaluate the impact of different exercise doses on hypertensive patients and to investigate whether high adherence to ACSM recommendations provides better treatment outcomes compared to interventions with low or uncertain adherence. This study aims to explore suitable exercise doses for the prevention and management of hypertension.

## Materials and methods

### Data sources and searches

We searched PubMed, Embase, Web of Science, and Cochrane databases from the date of establishment to July 16, 2024. The search strategy focused on the impact of exercise on hypertensive patients and followed the PICOS principle, which was mainly related to research participants, interventions, and research methods. The search included the following subject headings and keywords: (“Hypertension” or “Blood Pressure, High” or “Blood Pressures, High” or “High Blood Pressure” or “High Blood Pressures” or “Systolic blood pressure” or “Diastolic pressure” or “Hypertensive disease” or “Primary hypertension” or “Secondary hypertension” or “Essential hypertension” or “Endocrine hypertension”) AND (“Exercise” or “Exercises” or “Sports” or “Trainings” or “Fitness” or “Physical exercise” or “Physical activity” or “Physical fitness” or “Aerobic exercise” or “Resistance training” or “Strength training” or “Running” or “Tai Chi” or “Jogging” or “Bicycling” or “Motor activity” or “Physical workout”) AND (“Randomized controlled trial” or “controlled clinical trial” or “randomized” or “placebo” or “randomly”). The search strategy was provided in S1 Appendix.

### Inclusion and exclusion criteria of studies

Studies were included if they met the following criteria: (a) published randomized controlled trials; (b) study subjects with hypertension or prehypertension; (c) the intervention for the experimental group could be any type of exercise; (d) the control group received no treatment or treatments not related to exercise; (e) none of the participants had received specific pharmacological treatments; (f) outcome measures in the study included systolic blood pressure (SBP), diastolic blood pressure (DBP), and heart rate (HR).

Studies were excluded if they met the following criteria: (a) studies reported as review or conference abstracts; (b) the control group received exercise as the intervention; (c) study subjects had cardiovascular diseases; (d) study subjects were on specific medications during the exercise intervention; (e) the intervention period was less than four weeks.

### Data screening and extraction

Two authors (JC and WLC) independently screened the retrieved articles based on the inclusion and exclusion criteria. Initially, they reviewed the titles and abstracts of the articles. If either author deemed that a study met the inclusion criteria, the full text of the article was obtained. The two authors then independently assessed the full text to determine if it met the inclusion standards. In cases of disagreement, a third author (JX) was consulted to discuss and reach a consensus. There were no restrictions on the age, gender, cultural level, income, region, body mass index, publication date, or language.

The data extraction process was performed independently by two authors (JC and WLC). An Excel spreadsheet was designed to extract relevant data from the included studies, including primary outcome measures (SBP, DBP, HR), basic information (title, author names, publication year, and country), participant information (age, gender, sample size, and disease duration), intervention details (type of exercise, exercise frequency, exercise intensity, and exercise duration), and risk assessment features.

After data extraction, the exercise intervention dose and adherence were evaluated. The exercise doses in the included studies were evaluated based on the ACSM recommendations for hypertension (Table 1). Two authors (JC and WLC) independently scored each exercise intervention according to ACSM recommendations (including exercise frequency, intensity, duration, etc.), in order to assess the adherence to the exercise doses.

**Table 1:** ACSM exercise recommendations for hypertensive patients.

Exercise Dose	Aerobic Exercise	Resistance Exercise	Flexibility Exercise
Frequency	5–7 days/week	2–3 days/week	≥ 2–3 days per week
Intensity	Moderate intensity (40%-59% reserve oxygen uptake VO2R or reserve heart rate HRR); RPE of 12–13 on the 6–20 scale	Start with 60%-70% of 1RM and gradually increase to 80% of 1RM. Perform at least 8–10 different kinds of exercises. Muscular strength: 8~10 repetitions per set; muscular endurance: 12–20 repetitions per set	Full range of flexion, extension, and rotation, or stretching to the point of feeling tension or slight discomfort
Time	Cumulative or continuous exercise of ≥30 min/day; if done in separate sessions, each session should not be less than 10 min	Muscular Strength: 2–4 sets of repetitions	Static stretching hold 10-30s, repeat each movement 2–4 times
		Muscular Endurance: ≤2 sets of repetitions	

HRR: Reserve heart rate. VO^2^R: reserve oxygen uptake. RPE: Rating of Perceived Exertion.1RM: Maximum power of 1 repetition.

The scoring range for each exercise parameter was 0 to 2 points. A score of 2 points indicated that the parameter met the standard; a score of 1 point indicated uncertainty; and a score of 0 points indicated non-compliance with the standard. In cases of disagreement between the two authors, a third author was consulted to reach a final consensus. Based on this scoring system, we calculated the proportion of exercise dose adherence to the ACSM recommendations for hypertension. A proportion of ≥70% was classified as high adherence to ACSM recommendations, while a proportion <70% was classified as low or uncertain adherence to ACSM recommendations.

### Statistical analysis

Meta-analysis was analyzed using STATA 16.0. The studies were divided into two groups, one representing high adherence to ACSM recommendations and the other representing low or uncertain adherence. The heterogeneity among studies within each subgroup was assessed using Higgins I^2^ statistics and interpreted according to the Cochrane Handbook guidelines [22]. When assessing heterogeneity, if I^2^ ≤ 50%, a fixed-effect model was used; if I^2^ > 50%, a random-effects model was applied. Effect sizes were represented as standardized mean differences (SMD) with 95% confidence intervals (CI). Publication bias was assessed by constructing funnel plots of the effect sizes and standard errors for each study. The funnel plots were evaluated using Begg’s rank correlation test, with P < 0.05 considered statistically significant. Sensitivity analysis was performed by sequentially excluding studies to test the robustness of the results. The effects of subgroup treatments were compared using Cochran’s Q test and Higgins’ I^2^ statistics, with P < 0.05 indicating statistically significant differences.

### Literature quality assessment

Two authors (JC and WLC) independently evaluated the quality of the included studies using the Risk of Bias tool (Rob 2) [23] based on the Cochrane systematic review standards for randomized controlled trials. The Rob 2 tool includes random sequence generation, allocation concealment, blinding of participants and researchers, blinding of outcome assessment, incomplete outcome data, selective reporting, and other sources of bias. According to the Cochrane Handbook, reviewers rated each domain as “low risk,” “some concerns,” and “high risk”. In cases of disagreement during the scoring process, a third author (JX) was consulted to resolve the issue through discussion.

## Results

### Literature search and selection

**Fig 1:**
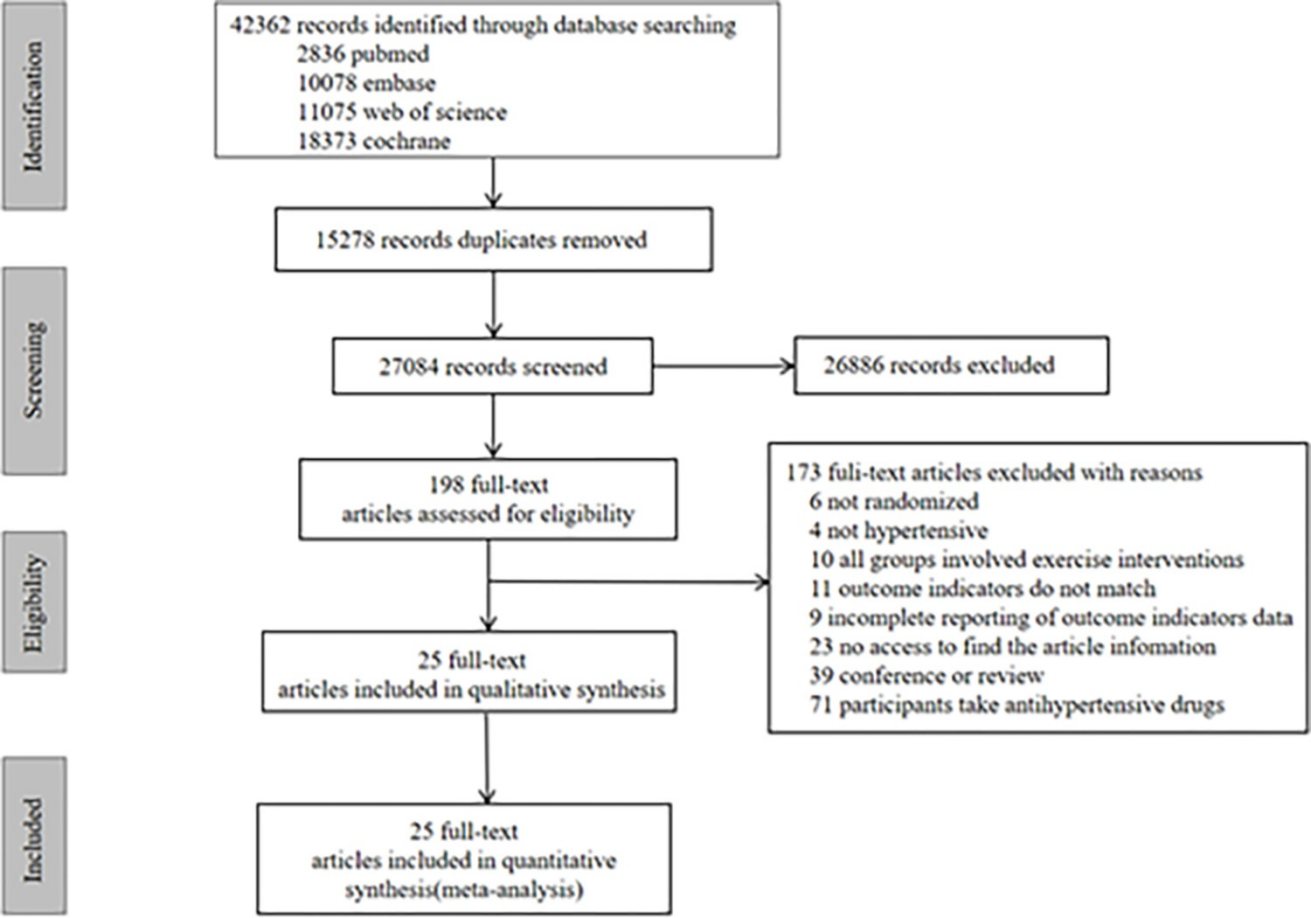
Flowchart of PRISMA study.

According to the literature search strategy, a total of 42,362 articles were retrieved from four databases. After removing 15,278 duplicate articles, 27,084 articles remained. After reviewing titles and abstracts, 198 articles were selected. Ultimately, 25 studies were included based on the inclusion and exclusion criteria [24–48] (Fig 1).

### Description of the characteristics of the studies

The 25 included articles covered 29 comparative research studies, with a total of 1,678 participants, including 838 participants in the experimental group and 840 participants in the control group. Four studies involved two exercise intervention groups. The reported ages of participants ranged from 17.9 to 74.5 years. The hypertension levels and degrees among the recruited participants included prehypertension, stage 1 hypertension, stage 1–2 hypertension, mild hypertension, and mild-to-moderate hypertension. The studies were conducted in various countries, including Brazil, Ethiopia, Egypt, Japan, Norway, India, Iran, Switzerland, Thailand, South Africa, the United States, South Korea, and China (Table 2).

**Table 2:** Overview of general characteristics of the study and participants.

Author, Year	Nation	Sample Size		Gender Ratio				Age (years)		Disease Level
		T	C	T(M)	T(F)	C(M)	C(F)	T	C	
Mota. M. R.2013	Brazil	32	32	0	32	0	32	67.50±7.00	66.80±5.40	NR
Thiyagarajan, R.2014	India	51	49	31	20	31	18	44.08±9.42	42.47±9.00	Prehypertension
Molmen-Hansen, H. E.2012	Norway	25	25	15	10	14	11	52.50±7.40	51.30±9.20	Hypertension stage 1–2
Molmen-Hansen, H. E.2012	Norway	23	25	13	10	14	11	53.60±6.50	51.30±9.20	Hypertension stage 1–2
Tsai, J. C.2002	Taiwan, China	12	11	7	5	5	6	49.60±9.30	46.20±5.60	Mild Hypertension
Stewart, K. J.2005	USA	51	53	25	26	26	27	63.00±5.333	64.10±6.1676	Mild Hypertension
Taha, M. M.2016	Egypt	23	23	0	23	0	23	48.17±2.20	47.78±2.59	Mild Hypertension
Shou, X. L.2019	China	98	100	48	50	55	45	NR	NR	Hypertension stage 1
Knoepfli-Lenzin, C.2010	Switzerland	15	17	15	0	17	0	NR	NR	Mild hypertension
Knoepfli-Lenzin, C.2010	Switzerland	15	17	15	0	17	0	NR	NR	Mild hypertension
Lee, M. S.2004	South Korea	17	19	8	9	6	13	52.60±5.10	54.30±5.50	Mild to moderate hypertension
Songcharern, N.2022	Thailand	15	15	15	0	15	0	19.60±1.70	19.50±0.50	Prehypertension
Yakasai, A. M.2021	South Africa	17	16	NR	NR	NR	NR	40.30±15.50	40.10±18.10	Mild to moderate hypertension
Yakasai, A. M.2021	South Africa	19	17	NR	NR	NR	NR	43.10±20.10	40.07±11.80	Mild to moderate hypertension Hypertension
Lee, M. S.2003	South Korea	29	29	NR	NR	NR	NR	56.00± 5.90	56.50± 7.20	NR
Higashi, Y.1999	Japan	10	7	7	3	6	1	NR	NR	Mild hypertension
Tanaka, H.1997	USA	12	6	7	5	3	3	47.00±10.392	49.00±12.247	Hypertension stage 1–2
Tsai, J. C.2004	Taiwan, China	52	50	24	28	23	27	48.80±6.30	49.30±7.20	Mild to moderate hypertension
Tsai, J. C.2003	Taiwan, China	37	39	19	18	19	20	51.60±16.30	50.50±9.80	Hypertension stage 1
Park, J. E.2017	South Korea	25	27	13	12	22	5	54.52±6.96	52.93±8.45	Mild hypertension
Wong, A.2020	USA	14	14	0	14	0	14	22.00±3.7417	23.00±3.7417	NR
Son, W. M.2020	South Korea	10	10	0	10	0	10	67.70±1.00	67.40±1.10	Hypertension stage 1
Lin, B.2022	China	50	49	NR	NR	NR	NR	64.20±4.04	63.80±4.40	NR
Phoemsapthawee. J.2021	Thailand	10	10	10	0	10	0	20.50±1.20	20.60±1.10	Prehypertension
Nosrat- abad. T. H.2021	Iran	15	15	15	0	15	0	NR	NR	Prehypertension
Nosrat-abad, T. H.2021	Iran	15	15	0	15	0	15	NR	NR	Prehypertension
Singh, V. P.2022	India	117	121	NR	NR	NR	NR	NR	NR	Prehypertension
Daimo, M.2020	Ethiopia	12	12	12	0	12	0	38.42±4.69	37.60±3.60	Hypertension stage 1
Woramontri, Chutima.2024	Thailand	17	17	5	12	5	12	65.18+3.83	65.35+3.94	Hypertension stage 1

T: treatment group. C: control group. M: male. F: female. NR: not reported.

According to the reported outcome measures, all 29 studies included SBP and DBP, while 20 studies reported HR. The duration of the interventions ranged from 6 weeks to 6 months across the 29 studies. The frequency of exercise varied from 3 to 7 times per week. The interventions in the experimental groups included a variety of methods such as qigong, yoga, brisk walking, cycling, tai chi, running, soccer, HIIT, swimming, and Pilates. The control group participants did not receive any exercise intervention. According to ACSM recommendations, 27 studies involved aerobic exercise dose, 5 studies involved resistance exercise dose, and 17 studies involved flexibility exercise dose (Table 3).

**Table 3:** Characteristics of the study intervention.

Author, year	Interventions	Length of intervention	SBP	DBP	HR
Mota. M. R. 2013	Resistance Training	16weeks	√	√	
Thiyagarajan, R.2014	Yoga	12weeks	√	√	√
Molmen-Hansen, H. E.2012	Aerobic Interval Training	12weeks	√	√	√
Molmen-Hansen, H. E.2012	Moderate intensity continuous training	12weeks	√	√	√
Tsai, J. C.2002	Running	12weeks	√	√	√
Stewart, K. J.2005	Resistance and Aerobic	6months	√	√	√
Taha, M. M.2016	HIIT	10weeks	√	√	
Shou, X. L.2019	24-Style Simplified Taijiquan	3months	√	√	
Knoepfli-Lenzin, C.2010	Soccer	12weeks	√	√	√
Knoepfli-Lenzin, C.2010	Running	12weeks	√	√	√
Lee, M. S.2004	Qigong	8weeks	√	√	
Songcharern, N.2022	Aerobics and Resistance	8weeks	√	√	√
Yakasai, A. M.2021	Cycling	6weeks	√	√	√
Yakasai, A. M.2021	Cycling	6 weeks	√	√	√
Lee, M. S.2003	Qigong	10 weeks	√	√	√
Higashi, Y.1999	Brisk Walking	12 weeks	√	√	√
Tanaka, H.1997	Swimming	10weeks	√	√	√
Tsai, J. C.2004	Running	10weeks	√	√	√
Tsai, J. C.2003	Tai Chi	12weeks	√	√	√
Park, J. E.2017	Qigong	12weeks	√	√	
Wong, A.2020	Pilates MP	12weeks	√	√	√
Son, W. M.2020	Resistance Band Exercise Training	12weeks	√	√	
Lin, B.2022	Tai Chi	12weeks	√	√	
Phoemsapthawee, J.2021	Resistance and Aerobic	12weeks	√	√	√
Nosrat-abad, T. H.2021	Tai Chi	8weeks	√	√	√
Nosrat-abad, T. H.2021	Tai Chi	8weeks	√	√	√
Singh, V. P.2022	Yoga	16weeks	√	√	
Daimo, M.2020	Brisk Walking	16weeks	√	√	
Woramontri, Chutima.2024	Pilates MP	12weeks	√	√	√

### Methodological quality assessment

**Fig 2:**
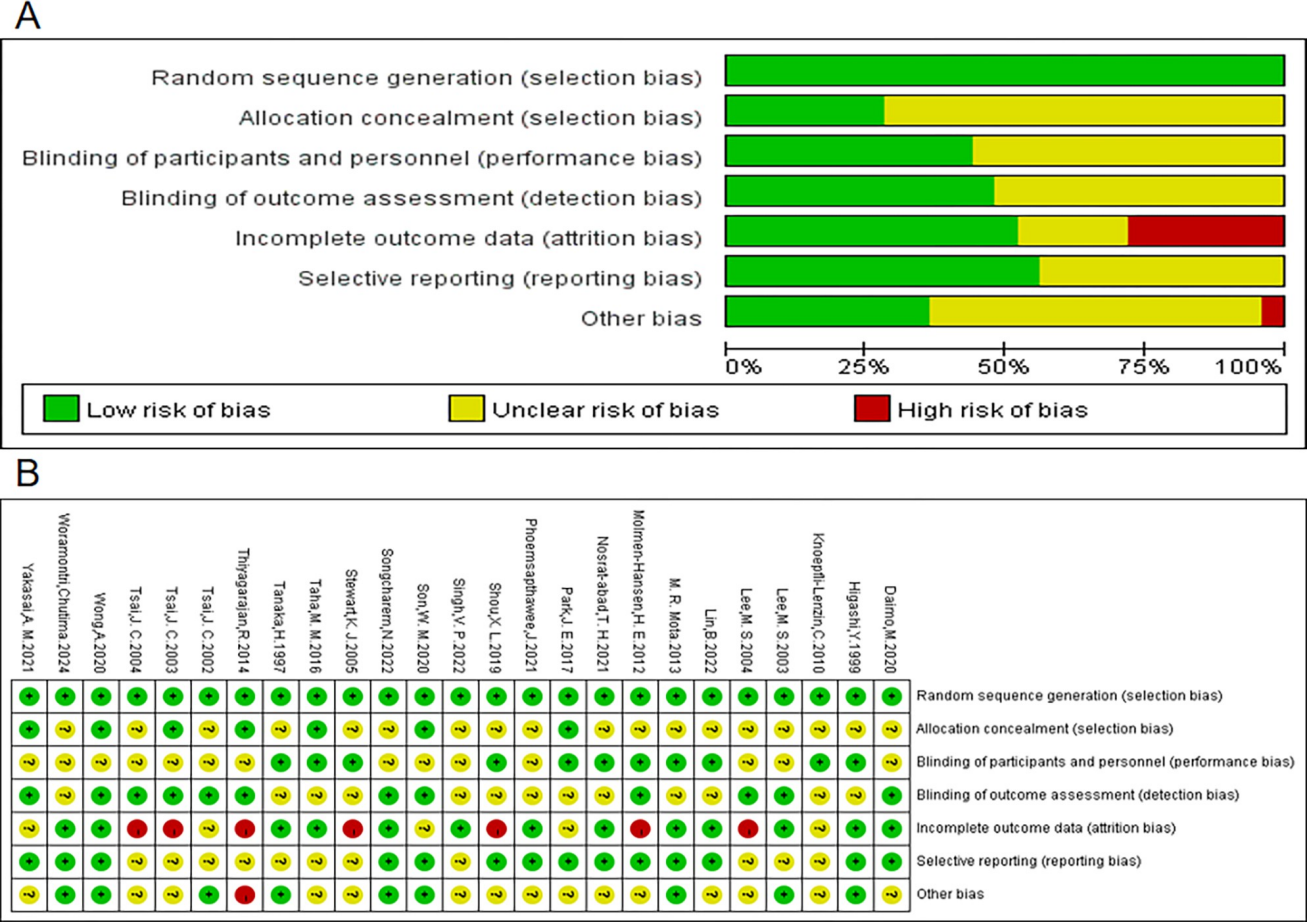
Authors’ assessment of risk of bias for each included study.

The risk of bias was assessed for all included studies. All 25 studies were randomized controlled trials. Regarding allocation concealment, 7 studies explicitly reported their allocation methods and were considered to have a low risk of bias. For blinding of participants and researchers, 14 studies were deemed to have an uncertain risk due to insufficient reporting on blinding procedures. In terms of outcome assessment blinding, 12 studies employed random testing, professional staff, or blinded assessors, and were considered to have a low risk of bias. For incomplete outcome reporting, 13 studies had post-intervention participant numbers consistent with or nearly consistent with baseline numbers, indicating a low risk of bias; 7 studies had a small number of participant withdrawals (5–10 people) and were considered to have some concerns; 5 studies showed a large discrepancy in participant numbers before and after (more than 10 people) and were considered to have a high risk of bias. Regarding selective reporting, 11 studies were considered to have some concerns due to a lack of registration details or inadequate explanation of participant dropouts (Fig 2A and 2B).

### Adherence to ACSM

Adherence to the ACSM recommendations was greater than 70% in 16 of the 29 studies, while in 13 studies, adherence to the ACSM recommendations was less than 70%. The reasons for low adherence included exercise interventions that did not align with ACSM recommendations, such as insufficient weekly exercise frequency or excessively high exercise intensity. Additionally, some studies lacked sufficiently detailed exercise protocols, making proper evaluation difficult (Table 4).

**Table 4:** Assessment of ACSM adherence.

Author, year	Aerobics exercise						Resistance exercise								Flexibility exercise						ACSM compliance	
	Frequency (day/week)(day/week)		Intensity /workload		Duration (min)		Frequency (day/week)		Intensity /workload		Repetition (times)		Sets (groups)		Frequency (day/week)		Intensity /workload		Duration		Points	Percent
Mota. M. R.2013							3		60–80%1RM	☺	12–16	☺	8–12	☹							6/8	75%
Thiyagarajan, R.2014	3	☹	NR	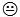	45	☺									3	☺	Full range of flexion	☺	NR	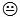	8/12	67%
Molmen-Hansen, H. E.2012	3	☹	60–95%HRmax	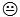	38	☺															3/6	50%
Molmen-Hansen, H. E.2012	3	☹	60%VO_2_max	☺	47	☺															4/6	67%
Tsai, J. C.2002	3	☹	60–70% HRmax	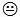	50	☺									3	☺	Total body stretch	☺	10min	☺	9/12	75%
Stewart, K. J.2005	3	☹	RPE18	☹	45	☺	3	☺	50%1RM	☹	10–15	☺	2	☺	3	☺	Total body stretch	☺	NR	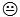	13/20	65%
Taha, M. M.2016	3	☹	70–85% HRmax	☹	40	☺															2/6	33%
Shou, X. L.2019	7	☺	50%-60% VO_2_max	☺	40–90	☺									7	☺	Full range of flexion	☺	2-4times	☺	12/12	100%
Knoepfli-Lenzin, C.2010	3	☹	65%HRmax	☹	60	☺															2/6	33%
Knoepfli-Lenzin, C.2010	3	☹	80% HRmax	☹	60	☺															2/6	33%
Lee, M. S.2004	2	☹	50–60%Max exercise capacity	☺	30	☺									2	☺	Full range of flexion	☺	NR	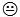	9/12	75%
Songcharern, N.2022	3	☹	50–70% HRR	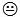	30	☺	3	☺	50–80%1RM	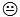	8–12	☺	2	☺	3	☺	Dynamic static stretching	☺	10min	☺	16/20	80%
Yakasai, A. M.2021	3	☹	ACSM	☺	30	☺															4/6	67%
Yakasai, A. M.2021	3	☹	ACSM	☺	30	☺															4/6	67%
Lee, M. S.2003	3	☹	50–60%Max exercise capacity	☺	30	☺									3	☺	Full range of flexion	☺	NR	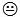	9/12	75%
Higashi, Y.1999	5–7次	☺	43~61% VO_2_max	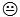	30	☺															5/6	83%
Tanaka, H.1997	3	☹	60% HRR	☺	60	☺															4/6	67%
Tsai, J. C.2004	3	☹	60–70% HRR	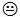	50	☺															3/6	50%
Tsai, J. C.2003	3	☹	64% HRmax	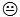	50	☺									3	☺	Full range of flexion	☺	10min	☺	9/12	75%
Park, J. E.2017	5	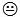	NR	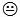	50	☺									5	☺	Full range of flexion	☺	NR	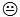	9/12	75%
Wong, A.2020	3	☹	NR	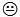	60	☺									3	☺	Full range of flexion	☺	10min	☺	9/12	75%
Son, W. M.2020							3	☺	40–80%1RM	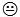	10–20	☺	2–4	☺	3	☺	Static Stretching	☺	20 min	☺	13/14	93%
Lin, B.2022	3	☹	NR	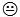	60	☺									3	☺	Full range of flexion	☺	15 min	☺	9/12	75%
Phoemsapthawee, J.2021	4	☹	50–70%HRR	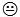	30	☺	4	☹	50–80%1RM	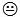	8–12	☺	2	☺	4	☺	Static Stretching	☺	15-30s	☺	14/20	70%
Nosrat-abad, T. H.2021	3	☹	NR	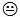	45	☺									3	☺	Full range of flexion	☺	10 min	☺	9/12	75%
Nosrat-abad, T. H.2021	3	☹	NR	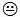	45	☺									3	☺	Full range of flexion	☺	10 min	☺	9/12	75%
Singh, V. P.2022	4	☹	NR	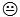	70	☺									4	☺	Full range of flexion	☺	NR	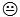	8/12	67%
Daimo, M.2020	3	☹	30–60%HRR	☺	30–45	☺															4/6	67%
Woramontri,Chutima.2024	3	☹	40–60%HRR	☺	30–45	☺									3	☺	Full range of flexion	☺	10 min	☺	10/12	83%

HRR: Reserve heart rate. VO^2^R: reserve oxygen uptake. RPE: Rating of Perceived Exertion.1RM: Maximum power of 1 repetition.NR: not reported. Happy/green face: fulfils recommendation (2 points), neutral/yellow face: uncertain fulfilment (1 point), unhappy/red face: does not fulfil recommendation (0 points).

Since all 29 studies included blood pressure indicators, 16 studies had high adherence to ACSM recommendations for SBP and DBP as outcome measures, while 13 studies had low or uncertain adherence. For studies with HR as the outcome measure, 10 studies had high adherence to ACSM recommendations, and 10 studies had low or uncertain adherence.

### Meta‑analysis

#### Analysis of SBP

**Fig 3:**
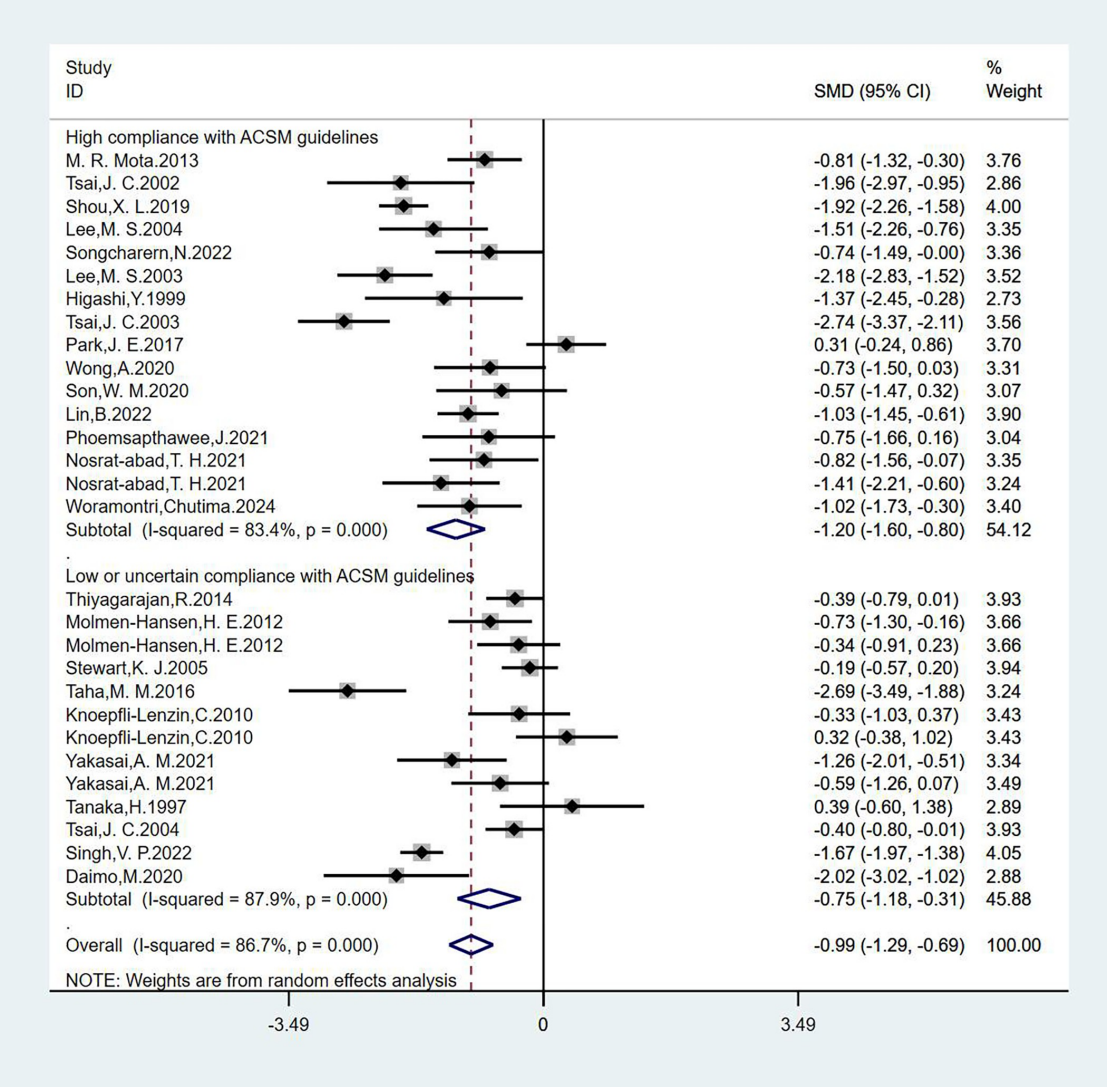
Forest plot of meta-analysis of the effect of exercise dose on SBP in hypertensive patients.

We analyzed 29 studies involving 1678 participants with SBP as the outcome measure, we found that the heterogeneity was greater than 50% by the heterogeneity test (I^2^ = 86.7%), so we used a random-effects model for statistical analysis. The overall combined SMD was −0.99 (95% CI: −1.29, −0.69), P = 0.000, indicating that exercise interventions are beneficial for SBP in hypertensive patients. In the subgroup analysis, we categorized the studies based on adherence to ACSM recommendations. The combined SMD for the subgroup with high ACSM adherence was −1.20 (95% CI: −1.60, −0.80), P = 0.000. For the subgroup with low or uncertain ACSM adherence, the combined SMD was −0.75 (95% CI: −1.18, −0.31), P = 0.000. The subgroup difference test revealed a significant difference between the interventions in studies with high ACSM adherence and those with low or uncertain ACSM adherence (Fig 3). Therefore, we conclude that exercise interventions with high adherence to ACSM recommendations have a superior therapeutic effect on SBP in hypertensive patients compared to those with low or uncertain adherence to ACSM recommendations.

#### Analysis of DBP

**Fig 4:**
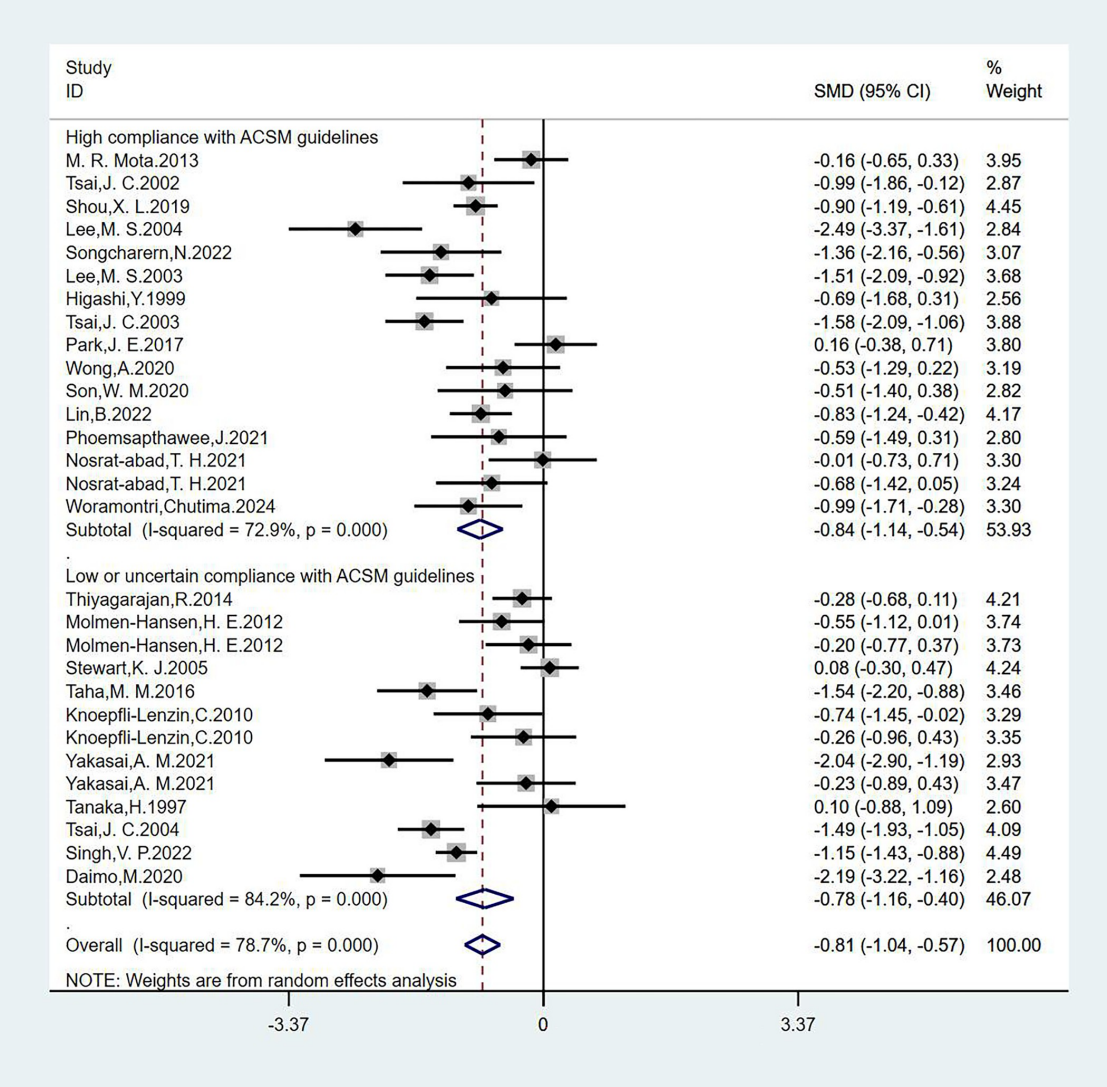
Forest plot of meta-analysis of the effect of exercise dose on DBP in hypertensive patients.

We analyzed 29 studies involving 1678 participants with DBP as the outcome measure, we found that the heterogeneity was greater than 50% by the heterogeneity test (I^2^ = 78.7%), so we used a random-effects model for statistical analysis. The overall combined SMD was −0.81 (95% CI: −1.04, −0.57), P = 0.000, indicating that exercise interventions are beneficial for DBP in hypertensive patients. In the subgroup analysis, we categorized the studies based on adherence to ACSM recommendations. The combined SMD for the subgroup with high ACSM adherence was −0.84 (95% CI: −1.14, −0.54), P = 0.000. For the subgroup with low or uncertain ACSM adherence, the combined SMD was −0.78 (95% CI: −1.16, −0.40), P = 0.000. The subgroup difference test revealed a significant difference between the interventions in studies with high ACSM adherence and those with low or uncertain ACSM adherence (Fig 4). Therefore, we conclude that exercise interventions with high adherence to ACSM recommendations have a superior therapeutic effect on DBP in hypertensive patients compared to those with low or uncertain adherence to ACSM recommendations.

#### Analysis of HR

**Fig 5:**
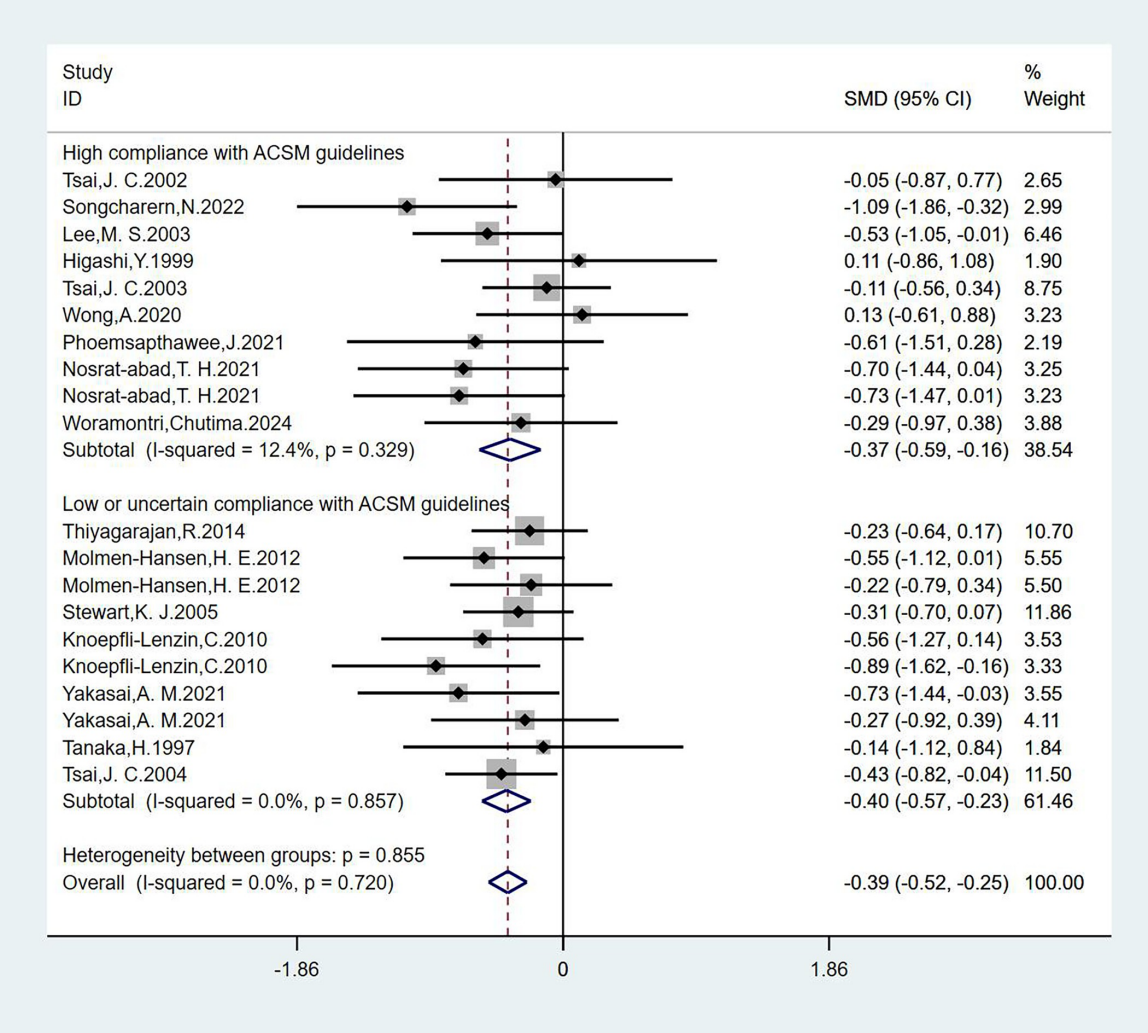
Forest plot of meta-analysis of the effect of exercise dose on HR in hypertensive patients.

We analyzed 20 studies involving 1178 participants with HR as the outcome measure, we found that the heterogeneity was less than 50% by the heterogeneity test (I^2^ = 0.0%), so we used a fixed effect model for statistical analysis. The overall combined SMD was −0.39 (95% CI: −0.52, −0.25), P = 0.720, indicating that exercise interventions do not show a significant effect on improving HR in hypertensive patients. In the subgroup analysis, we categorized the studies based on adherence to ACSM recommendations. The combined SMD for the subgroup with high ACSM adherence was −0.37 (95% CI: −0.59, −0.16), P = 0.329. For the subgroup with low or uncertain ACSM adherence, the combined SMD was −0.40 (95% CI: −0.57, −0.23), P = 0.857. The subgroup difference test showed that there was no significant difference between the interventions in studies with high ACSM adherence and those with low or uncertain ACSM adherence (Fig 5). Therefore, we conclude that exercise interventions with high adherence to ACSM recommendations do not have a superior effect on HR in hypertensive patients compared to interventions with low or uncertain adherence to ACSM recommendations.

**Fig 6:**
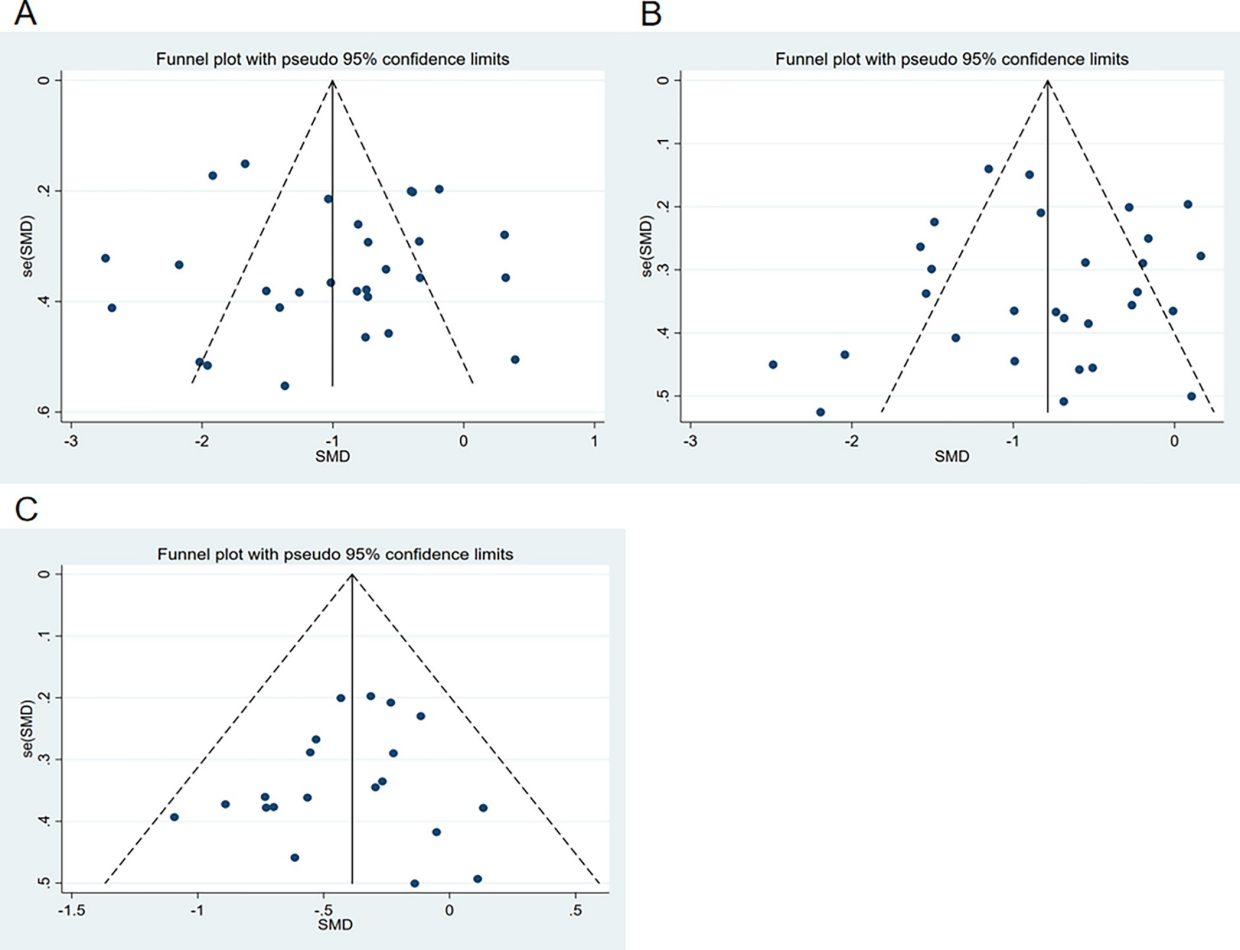
Funnel plot of meta-analysis of the effect of exercise dose on SBP, DBP, and HR in patients with hypertension.

**Fig 7:**
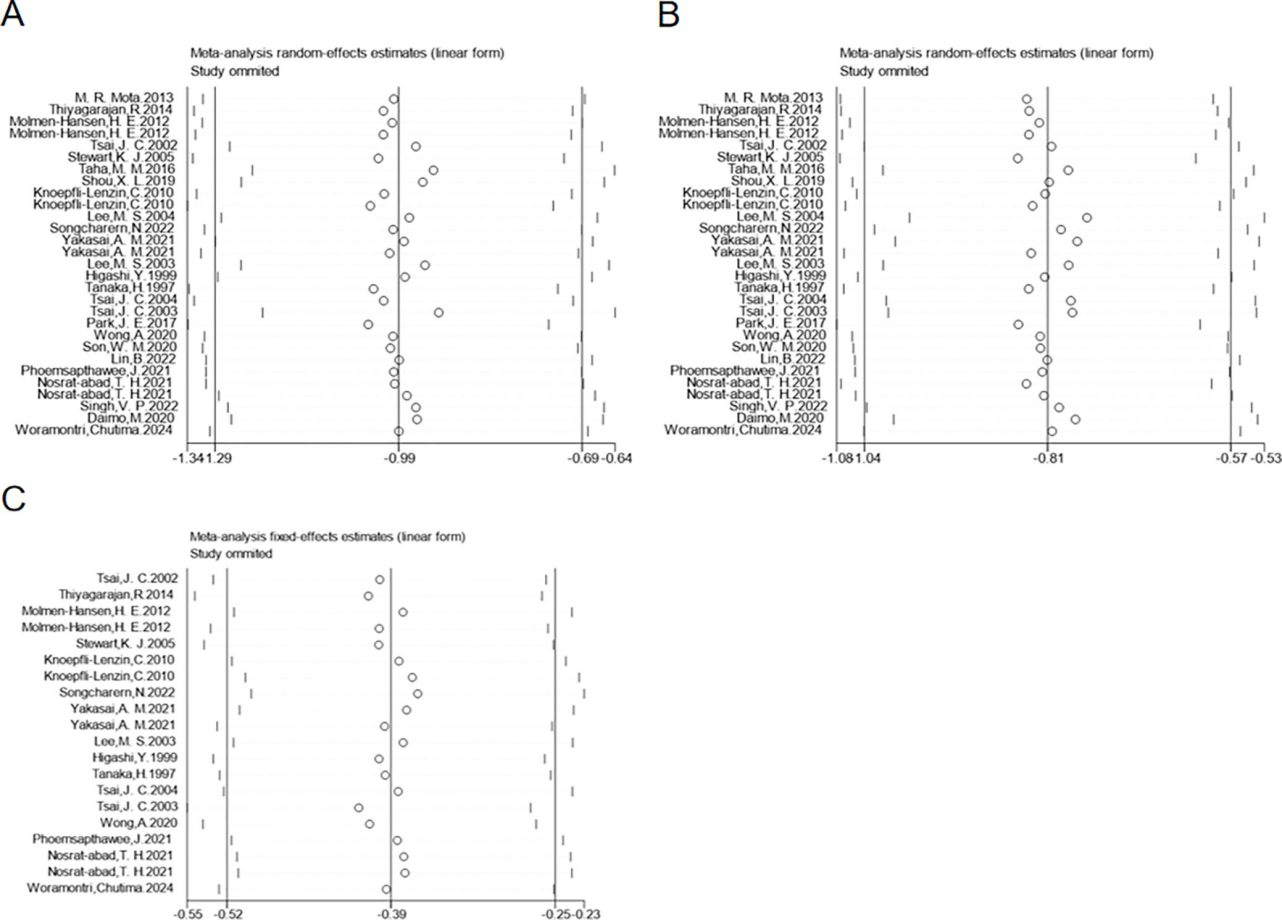
Sensitivity analysis of the effect of exercise dose on SBP, DBP, and HR in hypertensive patients.

We assessed publication bias for the outcomes of SBP, DBP, and HR using funnel plots (Fig 6A–6C). The plots showed approximate symmetry, indicating no significant publication bias. Further validation with Begg’s test resulted in P-values of 0.196, 0.378, and 0.581, respectively, confirming the absence of significant publication bias. Additionally, sensitivity analysis through the sequential removal of studies (Fig 7A–7C) revealed that no single study had a substantial impact on the overall results, suggesting that the findings were robust.

## Discussion

Our study found that exercise interventions with high adherence to ACSM recommendations were more effective in treating hypertension compared to those with low or uncertain adherence to ACSM recommendations. Based on ACSM recommendations for exercise in hypertensive patients, we conducted an extensive search for randomized controlled trials to evaluate the effects of various exercise doses on hypertension. Our meta-analysis reviewed 29 randomized controlled trials, involving 1,678 participants, assessing outcomes related to blood pressure (SBP, DBP) and HR. Both SBP and DBP were crucial targets in antihypertensive therapy, with SBP elevation being the most prevalent form of hypertension and a stronger predictor of hypertension risk [49]. Additionally, elevated HR has been associated with increased blood pressure and a higher risk of cardiovascular diseases [50]. Therefore, HR can also serve as an important predictor in assessing hypertension [51].

This study used ACSM adherence as a grouping factor to evaluate the effects of exercise dosage on improving heart rate and blood pressure in patients with hypertension. Subgroup analysis indicated that exercise interventions with high adherence to ACSM recommendations yielded better treatment outcomes for hypertension. The study further confirmed the positive impact of exercise interventions on patients with hypertension by incorporating various types of exercise. Prior studies have demonstrated that various types of exercise, such as aerobic exercise [52], resistance training [53], and endurance exercise[54], or different exercise modalities like Tai Chi [55, 56], yoga [57, 58], qigong [59, 60], and Ba Duan Jin [61, 62], can effectively lower blood pressure in hypertensive patients. Our study confirms this conclusion by incorporating multiple types of exercise interventions. In terms of the perspective of improvement in effect size (SMD): SBP (SMD = -0.99; 95% CI -1.30, -0.68), DBP (SMD = -0.80; 95% CI -1.04, -0.56), HR (SMD = -0.39; 95% CI -0.52, -0.25). This result demonstrates that various types of exercise, such as aerobic exercise, resistance training, and flexibility exercises, can effectively lower blood pressure. particularly with significant improvements in SBP, followed by DBP.

However, the results regarding the improvement in HR did not show a significant impact from adherence to ACSM recommendations. This finding was similar to a previous study by Ruangthai, which compared the effects of three exercise programs—ET, ST, and CBT, on hypertensive patients. The study found that all three exercise interventions could lower blood pressure, but there were no significant differences in HR changes among the groups [63]. Improvement in HR is vital for patients with hypertension, as elevated HR is closely linked to an increased risk of cardiovascular diseases. Effective HR control helps lower blood pressure and enhances heart health. The lack of significant improvement in HR observed in this study may be due to several factors, including insufficient intensity of the exercise interventions, individual differences (such as baseline HR, age, and gender), and the diversity of exercise types. This indicates that while exercise can enhance the overall health of hypertensive patients, the effectiveness of different interventions on HR improvement may vary. Future research should explore these factors in depth to help optimize exercise intervention strategies and more effectively improve HR control and overall health in patients with hypertension.

Lastly, this study had some limitations. First, as a meta-analysis that integrates existing research, it is inevitably influenced by the limitations and biases present in the original studies. Additionally, the heterogeneity in sample sizes and types of exercise interventions among the included studies may affect the overall results and interpretations. Future research could conduct network meta-analyses to explore the effects of different types of exercise on patients with hypertension, allowing for a more in-depth discussion of which exercise modalities or programs are most effective in improving hypertension. Another limitation was the lack of standardized protocols for exercise interventions and dosages in the included studies. Some randomized controlled trials provided incomplete descriptions of exercise measurements, which led us to employ a scoring assessment (0–2) to minimize biases arising from missing information. Detailed exercise prescriptions are crucial for determining the optimal exercise dosage, and future research and practice should focus on establishing more specific recommendations and personalized exercise prescriptions to help patients with hypertension better manage their blood pressure and HR. Finally, this study primarily focused on the effects of exercise on blood pressure and HR in patients with hypertension and did not explore related indicators such as blood lipid levels and quality of life.

To gain a more comprehensive understanding of the effects of exercise on patients with hypertension, future research should consider including a broader range of relevant outcome measures in the analysis. Additionally, we have observed that, apart from regular physical activity, non-pharmacological treatment options such as sodium restriction [64], weight loss [65], and low-calorie diets [66] all have demonstrated positive effects on lowering blood pressure in patients with hypertension. However, a common limitation across these studies is the lack of long-term outcome data for hypertensive patients. It remains uncertain whether blood pressure will remain well-controlled or continue to decrease after the cessation of interventions such as weight loss, sodium restriction, reduced alcohol intake, and regular physical exercise. Future research could benefit from larger sample sizes and longer follow-up periods in randomized controlled trials to address this question.

## Conclusions

In exploring appropriate exercise doses for hypertension patients, we found that exercise interventions with high adherence to ACSM recommendations had a more significant impact compared to those with low or uncertain adherence to ACSM recommendations. The analysis revealed that high adherence to ACSM recommendations was associated with the most notable improvements in SBP, followed by DBP and HR. This review supports that physical activity, as a non-pharmacological treatment, may effectively improve clinical outcomes for hypertension patients. Additionally, it underscores the importance of designing exercise programs according to ACSM recommendations doses for hypertension. Our findings reinforce this conclusion. However, given that some of the included studies had incomplete details on exercise interventions, future research with larger sample sizes and more detailed randomized controlled trials will be beneficial for further validation of these results.

## Supporting information

S1 Checklist PRISMA 2020 checklist. This checklist outlines the reporting standards followed in the systematic review and meta-analysis, ensuring compliance with the PRISMA 2020 guidelines.
(DOCX)

S1 Appendix Search strategy. This appendix outlines the detailed search strategy used to identify relevant studies for the systematic review, including the databases searched, keywords.
(DOCX)

S1 Data Minimal data set. This data file contains the essential data used in the analysis of the study, including key variables and measurements that support the findings presented in the manuscript.
(XLSX)

S1 Table Table listing all the studies identified in the literature search, including both the included and excluded studies. (XLSX)
