# Supplementary material for: Effect of exercise based on ACSM recommendations on blood pressure and heart rate in hypertensive patients: A systematic review and meta-analysis of randomized controlled trials
Source: PLOS Glob Public Health. 2024 Dec 16;4(12):e0003743. doi: 10.1371/journal.pgph.0003743 (PMC11649149; doi:10.1371/journal.pgph.0003743)
Supplement: S1 Appendix — This appendix outlines the detailed search strategy used to identify relevant studies for the systematic review, including the databases searched, keywords. (DOCX) [file pgph.0003743.s002.docx]

**S1 Appendix** Search Strategy

| Database | Search strategy | amount |
| --- | --- | --- |
| PubMed | | |
| #1 | Search: "Hypertension"[Mesh] Sort by: Most Recent | 325769 |
| #2 | Search: ((((((((((Blood Pressure, High[Title/Abstract]) OR (Blood Pressures, High[Title/Abstract])) OR (High Blood Pressure[Title/Abstract])) OR (High Blood Pressures[Title/Abstract])) OR (Systolic blood pressure[Title/Abstract])) OR (Diastolic pressure[Title/Abstract])) OR (Hypertensive disease[Title/Abstract])) OR (Primary hypertension[Title/Abstract])) OR (Secondary hypertension[Title/Abstract])) OR (Essential hypertension[Title/Abstract])) OR (Endocrine hypertension[Title/Abstract]) | 127180 |
| #3 | #1 OR #2 | 397359 |
| #4 | Search: "Exercise"[Mesh] Sort by: Most Recent | 258936 |
| #5 | Search: (((((((((((((((Exercises[Title/Abstract]) OR (Sports[Title/Abstract])) OR (Trainings[Title/Abstract])) OR (Fitness[Title/Abstract])) OR (Physical exercise[Title/Abstract])) OR (Physical activity[Title/Abstract])) OR (Physical fitness[Title/Abstract])) OR (Aerobic exercise[Title/Abstract])) OR (Resistance training[Title/Abstract])) OR (Strength training[Title/Abstract])) OR (Running[Title/Abstract])) OR (Tai chi[Title/Abstract])) OR (Jogging[Title/Abstract])) OR (Bicycling[Title/Abstract])) OR (Motor activity[Title/Abstract])) OR (Physical workout[Title/Abstract]) | 486937 |
| #6 | #4 OR #5 | 619960 |
| #7 | Search: ((((Randomized controlled trial [Publication Type]) OR (controlled clinical trial [Publication Type])) OR (randomized [Title/Abstract])) OR (placebo [Title/Abstract])) OR (randomly [Title/Abstract]) | 1405454 |
| #8 | #3 AND #6 AND #7 | 2836 |
| Embase | | |
| #1 | 'hypertension'/exp OR hypertension OR 'blood pressure, high':ab,ti OR 'blood pressures, high':ab,ti OR 'high blood pressure':ab,ti OR 'high blood pressures':ab,ti OR 'systolic blood pressure':ab,ti OR 'diastolic pressure':ab,ti OR 'hypertensive disease':ab,ti OR 'primary hypertension':ab,ti OR 'secondary hypertension':ab,ti OR 'essential hypertension':ab,ti OR 'endocrine hypertension':ab,ti | 1487895 |
| #2 | 'exercise'/exp OR exercise OR exercises:ab,ti OR sport:ab,ti OR trainings:ab,ti OR fitness:ab,ti OR exercise:ab,ti OR 'physical activity':ab,ti OR 'physical fitness':ab,ti OR 'aerobic exercise':ab,ti OR 'resistance training':ab,ti OR 'strength training':ab,ti OR running:ab,ti OR 'tai chi':ab,ti OR jogging:ab,ti OR bicycling:ab,ti OR 'motor activity':ab,ti OR 'physical workout':ab,ti | 1094276 |
| #3 | 'Randomized controlled trial:ab,ti OR 'controlled clinical trial':ab,ti OR 'randomly':ab,ti | 1576963 |
| #4 | #1 AND #2 AND #3 | 10078 |
| Web of Science | | |
| #1 | (((((((((((TS=(Hypertension)) OR TS= (Blood Pressure, High)) OR TS= (Blood Pressures, High)) OR TS= (High Blood Pressure)) OR TS= (High Blood Pressures)) OR TS= (Systolic blood pressure)) OR TS= (Diastolic pressure)) OR TS= (Hypertensive disease)) OR TS= (Primary hypertension)) OR TS= (Secondary hypertension)) OR TS= (Essential hypertension)) OR TS= (Endocrine hypertension) | 631288 |
| #2 | ((((((((((((((((TS=(Exercise)) OR TS=(Exercises)) OR TS=(Sports)) OR TS=(Trainings)) OR TS=(Fitness)) OR TS=(Physical exercise)) OR TS=(Physical activity)) OR TS=(Physical fitness)) OR TS=(Aerobic exercise)) OR TS=(Resistance training)) OR TS=(Strength training)) OR TS=(Running)) OR TS=(Tai chi)) OR TS=(Jogging)) OR TS=(Bicycling)) OR TS=(Motor activity)) OR TS=(Physical workout) | 2668198 |
| #3 | ((((TS=(Randomized controlled trial)) OR TS=(controlled clinical trial)) OR TS=(randomized)) OR TS=(placebo )) OR TS=(randomly) | 1646431 |
| #4 | #1 AND #2 AND #3 | 11075 |
| Cochrane | | |
| #1 | Hypertension):ti,ab,kw OR (Blood Pressure, High):ti,ab,kw OR (Blood Pressures, High):ti,ab,kw OR (High Blood Pressure):ti,ab,kw OR (High Blood Pressures):ti,ab,kw | 102565 |
| #2 | (Systolic blood pressure): ti,ab,kw OR (Diastolic pressure):ti,ab,kw OR (Hypertensive disease):ti,ab,kw OR (Primary hypertension):ti,ab,kw OR (Secondary hypertension):ti,ab,kw | 86797 |
| #3 | (Essential hypertension): ti,ab,kw OR (Endocrine hypertension):ti,ab,kw | 9655 |
| #4 | #1 OR #2 OR #3 | 126277 |
| #5 | (Exercise): ti,ab,kw OR (Exercises):ti,ab,kw OR (Sports):ti,ab,kw OR (Trainings):ti,ab,kw OR (Fitness):ti,ab,kw | 272144 |
| #6 | (Physical exercise): ti,ab,kw OR (Physical activity):ti,ab,kw OR (Physical fitness):ti,ab,kw OR (Aerobic exercise):ti,ab,kw OR (Resistance training):ti,ab,kw | 124813 |
| #7 | (Strength training): ti,ab,kw OR (Running):ti,ab,kw OR (Tai chi):ti,ab,kw OR (Jogging):ti,ab,kw OR (Bicycling):ti,ab,kw | 57463 |
| #8 | (Motor activity): ti,ab,kw OR (Physical workout):ti,ab,kw | 22149 |
| #9 | #5 OR #6 OR #7 OR #8 | 333415 |
| #10 | (Randomized controlled trial): ti,ab,kw OR (controlled clinical trial):ti,ab,kw OR (randomized):ti,ab,kw | 1422601 |
| #11 | #4 AND #9 AND #10 | 18373 |
